# 4-{2-[2-(4-Chloro­benzyl­idene)hydrazinyl­idene]-3,6-dihydro-2*H*-1,3,4-thia­diazin-5-yl}-3-(4-meth­oxy­phen­yl)sydnone

**DOI:** 10.1107/S1600536811013900

**Published:** 2011-04-22

**Authors:** Hoong-Kun Fun, Wan-Sin Loh, Nithinchandra, Balakrishna Kalluraya

**Affiliations:** a X-ray Crystallography Unit, School of Physics, Universiti Sains Malaysia, 11800 USM, Penang, Malaysia; b Department of Studies in Chemistry, Mangalore University, Mangalagangotri, Mangalore 574 199, India

## Abstract

The title compound, C_19_H_15_ClN_6_O_3_S, exists in *trans* and *cis* configurations with respect to the acyclic C=N bonds. The 3,6-dihydro-2*H*-1,3,4-thia­diazine ring adopts a half-boat conformation. The sydnone ring is approximately planar [maximum deviation = 0.013 (1) Å] and forms dihedral angles of 34.76 (4) and 48.67 (4)° with the benzene rings. An intra­molecular C—H⋯O hydrogen bond stabilizes the mol­ecular structure and forms an *S*(6) ring motif. In the crystal packing, inter­molecular N—H⋯N hydrogen bonds link centrosymmetrically related mol­ecules into dimers, generating *R*_2_^2^(8) ring motifs. The dimers are then linked into a three-dimensional network by inter­molecular C—H⋯O and C—H⋯Cl hydrogen bonds, and by C—H⋯π inter­actions. Further stabilization is provided by π–π inter­actions involving the sydnone rings, with centroid–centroid separations of 3.4198 (5) Å.

## Related literature

For background to and the biological activity of sydnones, see: Baker *et al.* (1949 ▶); Hedge *et al.* (2008 ▶); Rai *et al.* (2008 ▶); Kalluraya *et al.* (2003 ▶). For ring conformations, see: Cremer & Pople (1975 ▶). For related structures, see: Fun *et al.* (2010 ▶, 2011 ▶). For bond-length data, see: Allen *et al.* (1987 ▶). For hydrogen-bond motifs, see: Bernstein *et al.* (1995 ▶). For the stability of the temperature controller used in the data collection, see: Cosier & Glazer (1986 ▶).
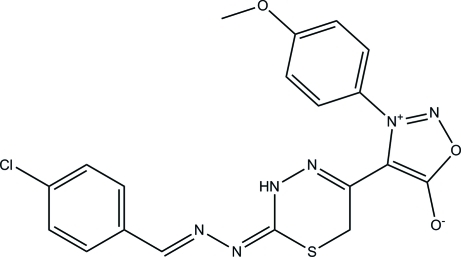


## Experimental

### 

#### Crystal data

C_19_H_15_ClN_6_O_3_S
*M* *_r_* = 442.88
Monoclinic, 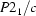

*a* = 7.2322 (2) Å
*b* = 22.7311 (6) Å
*c* = 12.9299 (3) Å
β = 114.426 (1)°
*V* = 1935.37 (9) Å^3^
*Z* = 4
Mo *K*α radiation
μ = 0.34 mm^−1^
*T* = 100 K
0.56 × 0.33 × 0.19 mm

#### Data collection

Bruker SMART APEXII DUO CCD area-detector diffractometer
Absorption correction: multi-scan (*SADABS*; Bruker, 2009) ▶ *T*_min_ = 0.832, *T*_max_ = 0.937
38523 measured reflections
10172 independent reflections
8764 reflections with *I* > 2σ(*I*)
*R*_int_ = 0.023

#### Refinement

*R*[*F* ^2^ > 2σ(*F* ^2^)] = 0.031
*wR*(*F* ^2^) = 0.088
*S* = 1.03
10172 reflections
272 parameters
H-atom parameters constrained
Δρ_max_ = 0.61 e Å^−3^
Δρ_min_ = −0.31 e Å^−3^

### 

Data collection: *APEX2* (Bruker, 2009 ▶); cell refinement: *SAINT* (Bruker, 2009 ▶); data reduction: *SAINT*; program(s) used to solve structure: *SHELXTL* (Sheldrick, 2008 ▶); program(s) used to refine structure: *SHELXTL*; molecular graphics: *SHELXTL*; software used to prepare material for publication: *SHELXTL* and *PLATON* (Spek, 2009 ▶).

## Supplementary Material

Crystal structure: contains datablocks global, I. DOI: 10.1107/S1600536811013900/rz2581sup1.cif

Structure factors: contains datablocks I. DOI: 10.1107/S1600536811013900/rz2581Isup2.hkl

Additional supplementary materials:  crystallographic information; 3D view; checkCIF report

## Figures and Tables

**Table 1:** Hydrogen-bond geometry (Å, °) *Cg*2 is the centroid of the N3/N4/C10/C9/S1 thia­diazine ring.

*D*—H⋯*A*	*D*—H	H⋯*A*	*D*⋯*A*	*D*—H⋯*A*
N3—H1⋯N2^i^	0.88	2.00	2.8841 (9)	174
C1—H1*A*⋯O2^ii^	0.93	2.59	3.4898 (10)	162
C9—H9*B*⋯O2	0.97	2.41	3.0433 (10)	123
C18—H18*A*⋯Cl1^iii^	0.93	2.77	3.6978 (7)	173
C19—H19*B*⋯*Cg*2^iv^	0.96	2.79	3.5792 (11)	140

Symmetry codes: (i) 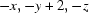; (ii) 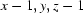; (iii) 
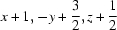
; (iv) 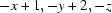.

**Fig. 1.**
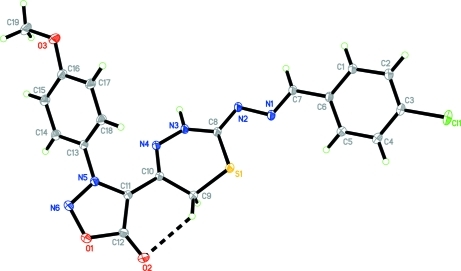
The molecular structure of the title compound, showing 50% probability displacement ellipsoids and the atom-numbering scheme. The dashed line indicates the intramolecular bond.

**Fig. 2.**
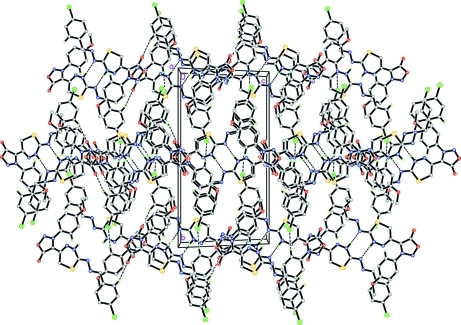
The crystal packing of the title compound, viewed along the a axis. H atoms not involved in the intermolecular interactions (dashed lines) have been omitted for clarity.

## supplementary crystallographic information

### Comment

Sydnones constitute a well defined class of mesoionic compounds consisting of the 1,2,3-oxadiazole ring system. The introduction of the concept of mesoionic structure for certain heterocyclic compounds in the year 1949 has proved to be a fruitful development in heterocyclic chemistry (Baker *et al.*, 1949). The study of sydnones still remains a field of interest because of their electronic structures and also because of the various types of biological activities displayed by some of them. Interest in sydnone derivatives has also been encouraged by the discovery that they exhibit various pharmacological activities (Hedge *et al.*, 2008; Rai *et al.*, 2008).

Encouraged by these reports and in continuation of our research for biologically active nitrogen containing heterocycles, a thiadiazine moiety at the 4-position of the phenylsydnone was introduced. The title compound was synthesized by the condensation of 4-bromoacetyl-3-arylsydnones with *N*'-[(4-chlorohlorophenyl)methylidene]thiocarbonohydrazide. 4-Bromoacetyl-3-arylsydnones were in turn obtained by the photochemical bromination of 4-acetyl-3-arylsydnones (Kalluraya *et al.*, 2003).

The title compound (Fig. 1) exists in *trans* and *cis* configurations with respect to the acyclic C7═N1 and C8═N2 bonds [C7═N1 = 1.2842 (9) Å and C8═N2 = 1.3061 (9) Å], respectively. The 3,6-dihydro-2*H*-1,3,4-thiadiazine ring (N3/N4/C10/C9/S1) adopts a half-boat conformation with the puckering parameters (Cremer & Pople, 1975), *Q* = 0.5322 (7) Å, Θ = 108.60 (8)°, φ = 136.74 (8)°. The sydnone ring (N5/N6/O1/C12/C11) is approximately planar with a maximum deviation of 0.013 (1) Å at atom C12 and forms dihedral angles of 34.76 (4) and 48.67 (4)° with the benzene rings (C1–C6 and C13–C18), respectively. An intramolecular C9—H9B···O2 hydrogen bond (Table 1) stabilizes the molecular structure and forms an *S*(6) ring motif (Bernstein *et al.*, 1995). Bond lengths (Allen *et al.*, 1987) and angles are within normal ranges and are comparable to the related structures (Fun *et al.*, 2010; Fun *et al.*, 2011).

In the crystal packing (Fig. 2), intermolecular N3—H1···N2 hydrogen bonds (Table 1) link centrosymmetrically related molecules to form dimers, generating *R*_2_^2^(8) ring motifs (Bernstein *et al.*, 1995). The dimers are then linked into a three-dimensional network by intermolecular C1—H1A···O2 and C18—H18A···Cl1 hydrogen bonds (Table 1) and stabilized by C—H···π interactions. The crystal structure is further consolidated by π–π interactions (Table 1), involving the sydnone rings (*Cg*1) with centroid-to-centroid separations *Cg*1···*Cg*1^v^ = 3.4198 (5) Å [symmetry code: (v) 2 - *x*,2 - *y*,1 - *z*].

### Experimental

To a solution of 4-bromoacetyl-3-(4-anisyl)sydnone (0.01 mol) and *N*'-(4-chlorophenyl)methylidene]thiocarbonohydrazide (0.01 mol) in ethanol, a catalytic amount of anhydrous sodium acetate was added. The solution was stirred at room temperature for 2 to 3 h. The solid product that separated out was filtered and dried. It was then recrystallized from ethanol. Crystals suitable for X-ray analysis were obtained by slow evaporation of a DMF/ethanol solution (1:2 *v*/*v*).

### Refinement

H1 was located from the difference Fourier map and was fixed at its found position with *U*_iso_(H) = 1.2 *U*_eq_(N) [N–H = 0.88 Å]. The remaining H atoms were positioned geometrically and refined using a riding model with C–H = 0.93–0.97 Å and with *U*_iso_(H) = 1.2 *U*_eq_(C) or 1.5 *U*_eq_(C) for methyl H atoms. A rotating group model was applied to the methyl group.

### Crystal data

**Table:**

C_19_H_15_ClN_6_O_3_S	*F*(000) = 912
*M**_r_* = 442.88	*D*_x_ = 1.520 Mg m^−^^3^
Monoclinic, *P*2_1_/*c*	Mo *K*α radiation, λ = 0.71073 Å
Hall symbol: -P 2ybc	Cell parameters from 9060 reflections
*a* = 7.2322 (2) Å	θ = 3.5–37.6°
*b* = 22.7311 (6) Å	µ = 0.34 mm^−^^1^
*c* = 12.9299 (3) Å	*T* = 100 K
β = 114.426 (1)°	Block, red
*V* = 1935.37 (9) Å^3^	0.56 × 0.33 × 0.19 mm
*Z* = 4	

### Data collection

**Table:**

Bruker SMART APEXII DUO CCD area-detector diffractometer	10172 independent reflections
Radiation source: fine-focus sealed tube	8764 reflections with *I* > 2σ(*I*)
graphite	*R*_int_ = 0.023
φ and ω scans	θ_max_ = 37.6°, θ_min_ = 1.8°
Absorption correction: multi-scan (*SADABS*; Bruker, 2009)	*h* = −12→12
*T*_min_ = 0.832, *T*_max_ = 0.937	*k* = −37→38
38523 measured reflections	*l* = −22→22

### Refinement

**Table:**

Refinement on *F*^2^	Primary atom site location: structure-invariant direct methods
Least-squares matrix: full	Secondary atom site location: difference Fourier map
*R*[*F*^2^ > 2σ(*F*^2^)] = 0.031	Hydrogen site location: inferred from neighbouring sites
*wR*(*F*^2^) = 0.088	H-atom parameters constrained
*S* = 1.03	*w* = 1/[σ^2^(*F*_o_^2^) + (0.0455*P*)^2^ + 0.4618*P*] where *P* = (*F*_o_^2^ + 2*F*_c_^2^)/3
10172 reflections	(Δ/σ)_max_ = 0.003
272 parameters	Δρ_max_ = 0.61 e Å^−^^3^
0 restraints	Δρ_min_ = −0.31 e Å^−^^3^

### Special details

**Table:**

Experimental. The crystal was placed in the cold stream of an Oxford Cryosystems Cobra open-flow nitrogen cryostat (Cosier & Glazer, 1986) operating at 100.0 (1) K.
Geometry. All e.s.d.'s (except the e.s.d. in the dihedral angle between two l.s. planes) are estimated using the full covariance matrix. The cell e.s.d.'s are taken into account individually in the estimation of e.s.d.'s in distances, angles and torsion angles; correlations between e.s.d.'s in cell parameters are only used when they are defined by crystal symmetry. An approximate (isotropic) treatment of cell e.s.d.'s is used for estimating e.s.d.'s involving l.s. planes.
Refinement. Refinement of *F*^2^ against ALL reflections. The weighted *R*-factor *wR* and goodness of fit *S* are based on *F*^2^, conventional *R*-factors *R* are based on *F*, with *F* set to zero for negative *F*^2^. The threshold expression of *F*^2^ > σ(*F*^2^) is used only for calculating *R*-factors(gt) *etc*. and is not relevant to the choice of reflections for refinement. *R*-factors based on *F*^2^ are statistically about twice as large as those based on *F*, and *R*- factors based on ALL data will be even larger.

### Fractional atomic coordinates and isotropic or equivalent isotropic displacement parameters (Å^2^)

**Table:**

	*x*	*y*	*z*	*U*_iso_*/*U*_eq_	
Cl1	−0.20673 (3)	0.599009 (8)	−0.296760 (19)	0.02044 (4)	
S1	0.27993 (3)	0.865504 (7)	0.177439 (15)	0.01416 (4)	
O1	0.86209 (8)	0.99555 (2)	0.58612 (4)	0.01533 (9)	
O2	0.69250 (10)	0.90827 (3)	0.56018 (5)	0.01973 (10)	
O3	0.62847 (10)	1.19710 (2)	0.08415 (5)	0.01920 (10)	
N1	−0.02123 (10)	0.86024 (3)	−0.03458 (5)	0.01436 (10)	
N2	0.00267 (10)	0.91926 (3)	−0.00409 (5)	0.01412 (10)	
N3	0.16911 (9)	0.98004 (3)	0.14755 (5)	0.01379 (9)	
H1	0.1098	1.0093	0.1006	0.017*	
N4	0.34093 (9)	0.99876 (3)	0.23756 (5)	0.01274 (9)	
N5	0.72206 (9)	1.03311 (3)	0.42028 (5)	0.01207 (9)	
N6	0.86571 (10)	1.04359 (3)	0.52215 (5)	0.01467 (10)	
C1	−0.20026 (12)	0.77461 (3)	−0.29399 (6)	0.01656 (12)	
H1A	−0.2271	0.8050	−0.3464	0.020*	
C2	−0.22174 (12)	0.71639 (3)	−0.33105 (6)	0.01765 (12)	
H2A	−0.2614	0.7077	−0.4075	0.021*	
C3	−0.18281 (11)	0.67161 (3)	−0.25156 (6)	0.01502 (11)	
C4	−0.12243 (11)	0.68343 (3)	−0.13639 (6)	0.01594 (11)	
H4A	−0.0979	0.6529	−0.0845	0.019*	
C5	−0.09958 (11)	0.74165 (3)	−0.10043 (6)	0.01524 (11)	
H5A	−0.0579	0.7501	−0.0237	0.018*	
C6	−0.13877 (11)	0.78790 (3)	−0.17873 (6)	0.01341 (10)	
C7	−0.11200 (11)	0.84940 (3)	−0.14139 (6)	0.01443 (11)	
H7A	−0.1597	0.8799	−0.1938	0.017*	
C8	0.13666 (10)	0.92500 (3)	0.10070 (6)	0.01224 (10)	
C9	0.37326 (12)	0.89915 (3)	0.31690 (6)	0.01527 (11)	
H9A	0.2663	0.8990	0.3437	0.018*	
H9B	0.4857	0.8761	0.3696	0.018*	
C10	0.44244 (10)	0.96112 (3)	0.31486 (6)	0.01225 (10)	
C11	0.62297 (10)	0.98132 (3)	0.41035 (6)	0.01216 (10)	
C12	0.71662 (11)	0.95431 (3)	0.51973 (6)	0.01398 (11)	
C13	0.69885 (10)	1.07654 (3)	0.33459 (6)	0.01235 (10)	
C14	0.68466 (12)	1.13502 (3)	0.35876 (6)	0.01581 (11)	
H14A	0.6883	1.1460	0.4289	0.019*	
C15	0.66483 (12)	1.17767 (3)	0.27707 (7)	0.01669 (12)	
H15A	0.6581	1.2173	0.2928	0.020*	
C16	0.65519 (11)	1.16009 (3)	0.17168 (6)	0.01458 (11)	
C17	0.67298 (12)	1.10052 (3)	0.14935 (6)	0.01655 (12)	
H17A	0.6690	1.0892	0.0793	0.020*	
C18	0.69642 (12)	1.05866 (3)	0.23103 (6)	0.01542 (11)	
H18A	0.7104	1.0191	0.2172	0.019*	
C19	0.61977 (13)	1.25837 (3)	0.10431 (8)	0.02065 (13)	
H19A	0.5968	1.2798	0.0361	0.031*	
H19B	0.7459	1.2707	0.1638	0.031*	
H19C	0.5108	1.2659	0.1266	0.031*	

### Atomic displacement parameters (Å^2^)

**Table:**

	*U*^11^	*U*^22^	*U*^33^	*U*^12^	*U*^13^	*U*^23^
Cl1	0.02240 (8)	0.01084 (7)	0.03075 (9)	−0.00245 (5)	0.01366 (7)	−0.00500 (6)
S1	0.01632 (7)	0.00916 (6)	0.01430 (7)	0.00094 (5)	0.00364 (6)	−0.00035 (5)
O1	0.0158 (2)	0.0165 (2)	0.01175 (19)	0.00025 (17)	0.00369 (17)	0.00065 (16)
O2	0.0252 (3)	0.0156 (2)	0.0162 (2)	−0.00022 (19)	0.0064 (2)	0.00420 (18)
O3	0.0257 (3)	0.0128 (2)	0.0186 (2)	−0.00033 (19)	0.0087 (2)	0.00338 (18)
N1	0.0161 (2)	0.0102 (2)	0.0146 (2)	−0.00112 (18)	0.00425 (19)	−0.00205 (17)
N2	0.0163 (2)	0.0099 (2)	0.0132 (2)	−0.00053 (18)	0.00306 (19)	−0.00127 (17)
N3	0.0150 (2)	0.0094 (2)	0.0127 (2)	0.00004 (17)	0.00144 (18)	−0.00074 (17)
N4	0.0135 (2)	0.0108 (2)	0.0118 (2)	−0.00057 (17)	0.00305 (18)	−0.00070 (17)
N5	0.0124 (2)	0.0115 (2)	0.0114 (2)	−0.00024 (17)	0.00396 (17)	−0.00076 (17)
N6	0.0152 (2)	0.0152 (2)	0.0117 (2)	−0.00103 (19)	0.00366 (19)	−0.00106 (18)
C1	0.0208 (3)	0.0119 (3)	0.0134 (3)	−0.0007 (2)	0.0035 (2)	−0.0005 (2)
C2	0.0218 (3)	0.0133 (3)	0.0156 (3)	−0.0015 (2)	0.0056 (2)	−0.0025 (2)
C3	0.0144 (3)	0.0103 (2)	0.0202 (3)	−0.0011 (2)	0.0071 (2)	−0.0021 (2)
C4	0.0176 (3)	0.0116 (2)	0.0188 (3)	0.0002 (2)	0.0077 (2)	0.0014 (2)
C5	0.0175 (3)	0.0125 (2)	0.0146 (3)	0.0001 (2)	0.0056 (2)	0.0005 (2)
C6	0.0137 (3)	0.0105 (2)	0.0137 (2)	−0.00017 (19)	0.0034 (2)	−0.00092 (19)
C7	0.0159 (3)	0.0108 (2)	0.0137 (2)	−0.0004 (2)	0.0032 (2)	−0.00090 (19)
C8	0.0131 (2)	0.0100 (2)	0.0127 (2)	−0.00038 (19)	0.0044 (2)	−0.00029 (18)
C9	0.0185 (3)	0.0112 (2)	0.0134 (2)	−0.0021 (2)	0.0039 (2)	0.0007 (2)
C10	0.0132 (2)	0.0105 (2)	0.0120 (2)	−0.00038 (19)	0.0041 (2)	−0.00019 (18)
C11	0.0130 (2)	0.0108 (2)	0.0116 (2)	0.00004 (19)	0.00400 (19)	0.00039 (18)
C12	0.0148 (3)	0.0137 (3)	0.0122 (2)	0.0012 (2)	0.0045 (2)	0.00047 (19)
C13	0.0139 (2)	0.0103 (2)	0.0126 (2)	−0.00055 (19)	0.0052 (2)	−0.00006 (19)
C14	0.0207 (3)	0.0117 (2)	0.0157 (3)	−0.0002 (2)	0.0082 (2)	−0.0019 (2)
C15	0.0216 (3)	0.0106 (2)	0.0184 (3)	0.0005 (2)	0.0088 (2)	−0.0007 (2)
C16	0.0156 (3)	0.0116 (2)	0.0162 (3)	−0.0006 (2)	0.0061 (2)	0.0010 (2)
C17	0.0238 (3)	0.0123 (3)	0.0157 (3)	−0.0014 (2)	0.0103 (2)	−0.0010 (2)
C18	0.0219 (3)	0.0107 (2)	0.0153 (3)	−0.0008 (2)	0.0094 (2)	−0.0013 (2)
C19	0.0221 (3)	0.0127 (3)	0.0274 (4)	−0.0001 (2)	0.0105 (3)	0.0042 (2)

### Geometric parameters (Å, °)

**Table:**

Cl1—C3	1.7353 (7)	C4—C5	1.3897 (10)
S1—C8	1.7426 (7)	C4—H4A	0.9300
S1—C9	1.8125 (7)	C5—C6	1.4047 (10)
O1—N6	1.3767 (8)	C5—H5A	0.9300
O1—C12	1.4055 (9)	C6—C7	1.4653 (9)
O2—C12	1.2145 (9)	C7—H7A	0.9300
O3—C16	1.3582 (9)	C9—C10	1.4988 (9)
O3—C19	1.4231 (10)	C9—H9A	0.9700
N1—C7	1.2842 (9)	C9—H9B	0.9700
N1—N2	1.3887 (8)	C10—C11	1.4514 (10)
N2—C8	1.3061 (9)	C11—C12	1.4295 (9)
N3—C8	1.3675 (9)	C13—C14	1.3790 (9)
N3—N4	1.3724 (8)	C13—C18	1.3924 (9)
N3—H1	0.8830	C14—C15	1.3968 (10)
N4—C10	1.2896 (9)	C14—H14A	0.9300
N5—N6	1.3188 (8)	C15—C16	1.3939 (10)
N5—C11	1.3564 (9)	C15—H15A	0.9300
N5—C13	1.4411 (9)	C16—C17	1.4017 (10)
C1—C2	1.3942 (10)	C17—C18	1.3788 (10)
C1—C6	1.4007 (10)	C17—H17A	0.9300
C1—H1A	0.9300	C18—H18A	0.9300
C2—C3	1.3902 (11)	C19—H19A	0.9600
C2—H2A	0.9300	C19—H19B	0.9600
C3—C4	1.3938 (11)	C19—H19C	0.9600
			
C8—S1—C9	97.36 (3)	C10—C9—H9A	109.3
N6—O1—C12	110.94 (5)	S1—C9—H9A	109.3
C16—O3—C19	117.09 (6)	C10—C9—H9B	109.3
C7—N1—N2	116.04 (6)	S1—C9—H9B	109.3
C8—N2—N1	110.05 (6)	H9A—C9—H9B	108.0
C8—N3—N4	126.06 (6)	N4—C10—C11	118.23 (6)
C8—N3—H1	116.0	N4—C10—C9	123.36 (6)
N4—N3—H1	111.2	C11—C10—C9	118.15 (6)
C10—N4—N3	118.51 (6)	N5—C11—C12	105.27 (6)
N6—N5—C11	114.74 (6)	N5—C11—C10	127.54 (6)
N6—N5—C13	115.93 (6)	C12—C11—C10	126.68 (6)
C11—N5—C13	129.25 (6)	O2—C12—O1	121.08 (6)
N5—N6—O1	104.71 (5)	O2—C12—C11	134.63 (7)
C2—C1—C6	120.77 (7)	O1—C12—C11	104.28 (6)
C2—C1—H1A	119.6	C14—C13—C18	121.78 (6)
C6—C1—H1A	119.6	C14—C13—N5	118.81 (6)
C3—C2—C1	118.77 (7)	C18—C13—N5	119.38 (6)
C3—C2—H2A	120.6	C13—C14—C15	119.45 (6)
C1—C2—H2A	120.6	C13—C14—H14A	120.3
C2—C3—C4	121.79 (6)	C15—C14—H14A	120.3
C2—C3—Cl1	119.07 (6)	C16—C15—C14	119.24 (6)
C4—C3—Cl1	119.13 (5)	C16—C15—H15A	120.4
C5—C4—C3	118.85 (6)	C14—C15—H15A	120.4
C5—C4—H4A	120.6	O3—C16—C15	124.71 (6)
C3—C4—H4A	120.6	O3—C16—C17	114.86 (6)
C4—C5—C6	120.73 (7)	C15—C16—C17	120.43 (6)
C4—C5—H5A	119.6	C18—C17—C16	120.11 (6)
C6—C5—H5A	119.6	C18—C17—H17A	119.9
C1—C6—C5	119.08 (6)	C16—C17—H17A	119.9
C1—C6—C7	119.79 (6)	C17—C18—C13	118.94 (6)
C5—C6—C7	121.11 (6)	C17—C18—H18A	120.5
N1—C7—C6	118.46 (6)	C13—C18—H18A	120.5
N1—C7—H7A	120.8	O3—C19—H19A	109.5
C6—C7—H7A	120.8	O3—C19—H19B	109.5
N2—C8—N3	117.95 (6)	H19A—C19—H19B	109.5
N2—C8—S1	121.64 (5)	O3—C19—H19C	109.5
N3—C8—S1	120.34 (5)	H19A—C19—H19C	109.5
C10—C9—S1	111.64 (5)	H19B—C19—H19C	109.5
			
C7—N1—N2—C8	−164.75 (7)	C13—N5—C11—C12	175.91 (6)
C8—N3—N4—C10	−31.43 (10)	N6—N5—C11—C10	171.22 (6)
C11—N5—N6—O1	−0.60 (8)	C13—N5—C11—C10	−11.96 (11)
C13—N5—N6—O1	−177.86 (5)	N4—C10—C11—N5	−12.74 (10)
C12—O1—N6—N5	1.94 (7)	C9—C10—C11—N5	172.88 (6)
C6—C1—C2—C3	0.59 (12)	N4—C10—C11—C12	157.77 (7)
C1—C2—C3—C4	−0.26 (11)	C9—C10—C11—C12	−16.60 (10)
C1—C2—C3—Cl1	−179.36 (6)	N6—O1—C12—O2	178.68 (7)
C2—C3—C4—C5	−0.40 (11)	N6—O1—C12—C11	−2.46 (7)
Cl1—C3—C4—C5	178.71 (6)	N5—C11—C12—O2	−179.41 (8)
C3—C4—C5—C6	0.72 (11)	C10—C11—C12—O2	8.38 (13)
C2—C1—C6—C5	−0.27 (11)	N5—C11—C12—O1	1.97 (7)
C2—C1—C6—C7	178.37 (7)	C10—C11—C12—O1	−170.25 (6)
C4—C5—C6—C1	−0.40 (11)	N6—N5—C13—C14	−49.70 (9)
C4—C5—C6—C7	−179.03 (7)	C11—N5—C13—C14	133.51 (8)
N2—N1—C7—C6	179.07 (6)	N6—N5—C13—C18	128.71 (7)
C1—C6—C7—N1	−167.55 (7)	C11—N5—C13—C18	−48.07 (10)
C5—C6—C7—N1	11.07 (11)	C18—C13—C14—C15	0.78 (11)
N1—N2—C8—N3	−175.58 (6)	N5—C13—C14—C15	179.15 (7)
N1—N2—C8—S1	7.51 (8)	C13—C14—C15—C16	1.44 (11)
N4—N3—C8—N2	−157.12 (7)	C19—O3—C16—C15	3.20 (11)
N4—N3—C8—S1	19.84 (9)	C19—O3—C16—C17	−176.93 (7)
C9—S1—C8—N2	−165.71 (6)	C14—C15—C16—O3	177.44 (7)
C9—S1—C8—N3	17.44 (6)	C14—C15—C16—C17	−2.43 (11)
C8—S1—C9—C10	−44.06 (6)	O3—C16—C17—C18	−178.67 (7)
N3—N4—C10—C11	−179.45 (6)	C15—C16—C17—C18	1.21 (12)
N3—N4—C10—C9	−5.39 (10)	C16—C17—C18—C13	0.99 (12)
S1—C9—C10—N4	44.63 (9)	C14—C13—C18—C17	−2.00 (11)
S1—C9—C10—C11	−141.30 (5)	N5—C13—C18—C17	179.63 (7)
N6—N5—C11—C12	−0.91 (8)		

### Hydrogen-bond geometry (Å, °)

**Table:**

Cg2 is the centroid of the N3/N4/C10/C9/S1 thiadiazine ring.

**Table:**

*D*—H···*A*	*D*—H	H···*A*	*D*···*A*	*D*—H···*A*
N3—H1···N2^i^	0.88	2.00	2.8841 (9)	174
C1—H1A···O2^ii^	0.93	2.59	3.4898 (10)	162
C9—H9B···O2	0.97	2.41	3.0433 (10)	123
C18—H18A···Cl1^iii^	0.93	2.77	3.6978 (7)	173
C19—H19B···Cg2^iv^	0.96	2.79	3.5792 (11)	140

Symmetry codes: (i) −*x*, −*y*+2, −*z*; (ii) *x*−1, *y*, *z*−1; (iii) *x*+1, −*y*+3/2, *z*+1/2; (iv) −*x*+1, −*y*+2, −*z*.
